# Characterization of Partial Discharges in Dielectric Oils Using High-Resolution CMOS Image Sensor and Convolutional Neural Networks

**DOI:** 10.3390/s24041317

**Published:** 2024-02-18

**Authors:** José Miguel Monzón-Verona, Pablo González-Domínguez, Santiago García-Alonso

**Affiliations:** 1 Electrical Engineering Department (DIE), University of Las Palmas de Gran Canaria, 35017 Las Palmas de Gran Canaria, Spain; pablo.gonzalez@ulpgc.es; 2 Institute for Applied Microelectronics, University of Las Palmas de Gran Canaria, 35017 Las Palmas de Gran Canaria, Spain; 3 Department of Electronic Engineering and Automatics (DIEA), University of Las Palmas de Gran Canaria, 35017 Las Palmas de Gran Canaria, Spain; santiago.garciaalonso@ulpgc.es

**Keywords:** partial discharges, mineral oils, CMOS image sensor, convolutional neural network, deep learning, non-destructive diagnosis

## Abstract

In this work, an exhaustive analysis of the partial discharges that originate in the bubbles present in dielectric mineral oils is carried out. To achieve this, a low-cost, high-resolution CMOS image sensor is used. Partial discharge measurements using that image sensor are validated by a standard electrical detection system that uses a discharge capacitor. In order to accurately identify the images corresponding to partial discharges, a convolutional neural network is trained using a large set of images captured by the image sensor. An image classification model is also developed using deep learning with a convolutional network based on a TensorFlow and Keras model. The classification results of the experiments show that the accuracy achieved by our model is around 95% on the validation set and 82% on the test set. As a result of this work, a non-destructive diagnosis method has been developed that is based on the use of an image sensor and the design of a convolutional neural network. This approach allows us to obtain information about the state of mineral oils before breakdown occurs, providing a valuable tool for the evaluation and maintenance of these dielectric oils.

## 1. Introduction

The measurement and detection of partial discharges (PDs) are invaluable tools for the evaluation of insulation quality, generating improvements in both maintenance practices and risk management in energy devices. A literature review on current online PD monitoring techniques used for different high-voltage electrical components in power electrical systems is presented in [1].

In [1], a detailed review and analysis of online monitoring of PDs in order to reduce breakdowns in electrical power systems and the advances in this area are presented. An intelligent framework based on wireless sensors is also proposed to improve the performance of power systems.

Ref. [2] focuses on researching and analyzing measurement techniques that use UHF to monitor PDs in high-voltage systems in real time, which could significantly contribute to the early detection of possible problems in the electrical system and its preventive maintenance. In [3], how voltage harmonics affect the diagnosis of PDs in electric motors that are powered by variable-frequency drives is addressed.

In [4], an analysis of the similarities and differences in surface PDs, both in direct current (DC) and alternating current (AC), is carried out. Furthermore, the underlying mechanisms that generate these discharges and the patterns associated with them are investigated in depth.

PDs in transformer oil play a fundamental role in the progressive deterioration of paper–oil insulation. The bubbles present in the transformer oil, the formation of which can be due to various factors, including some non-electrical, have the ability to move to areas of high electrical intensity due to the cooling system, potentially leading to PDs.

Despite their importance, the underlying mechanisms of PDs in fluids still lack in-depth investigation. Therefore, the purpose of this paper is to examine the behavior of PDs generated within gaseous bubbles dissolved in the dielectric oil when subjected to a high-field-intensity alternating electric field. This is achieved by using a low-cost, high-resolution CMOS image sensor and a neural network trained for PD detection.

Given the relevance of PDs originating from bubbles of gases dissolved in liquid dielectrics, a detailed analysis of the most significant works in this area of knowledge is carried out below.

In [5], the results of a numerical investigation that addressed the evolution of streamers in bubbles immersed in liquids are presented. It is established that the formation of streamers does not occur in very small bubbles unless the preexisting or injected charges are considerable enough to trigger PD.

In [6], a detailed analysis of the dynamics of bubbles inside dielectric oil is carried out. The study of the behavior of air bubbles that rise in the transformer oil under the influence of an alternating electric field is investigated, including the measurement of the degree of deformation of these bubbles.

The study presented in [7] focuses on the electrical breakdown voltage of transformer oil when it presents gaseous bubbles. The breakdown voltage stabilization effect is postulated to be due to the predominance of PDs in the sulfur hexafluoride bubbles, as opposed to the stochastic influence of chemical impurities and particulate materials.

In [8], a description is given of the different stages prior to collapse in the dielectric oil of a transformer. This is achieved by deforming an air bubble introduced between high-voltage (HV) electrodes, adjusting the separation between the electrodes and maintaining a non-uniform electric field, while keeping the hydrostatic pressure constant.

In [9], it is explored how the paper insulation system, in a transformer, retains a certain amount of moisture. Moisture tends to release from the paper as temperatures increase, being absorbed by the insulating oil. However, in situations of sudden temperature increase, such as when energizing the transformer for the first time or during an emergency overload, this moisture is abruptly released from the paper into the oil, causing gaseous bubbles.

In [10], the pattern of the evolution of bubbles in liquids in the presence of repetitive pulsed power is analyzed. Finally, ref. [11] addresses the mechanisms of bubble discharge and deterioration of paper–oil insulation, offering guidance for the diagnosis of transformer failures.

The use of low-cost camera systems in remote sensing applications is widely recognized. Research on the utilization of these low-cost cameras in engineering and science applications is discussed in detail in [12]. With a similar approach, ref. [13] focuses on the study of the corona effect in aeronautical environments using low-cost cameras, such as the Raspberry Pi.

A more contemporary analysis of low-cost, high-resolution image sensors is presented in [14]. This work explores the broad application of optical recording and tracking systems in mechanical and physical testing, such as strain measurements and high-speed particle tracking. These systems provide detailed and precise visual data that escape perception with the naked eye due to their speed.

The determination of breakdown voltage in mineral dielectric oils using low-cost image sensors is analyzed in [15]. This study provides a distribution of the Kelvin forces moments before the dynamic behavior of the electric arc begins, as well as the state of the gases that are generated moments after the appearance of the electric arc in the oil.

In [16], a set of hypersensitive optical sensors is developed for multispectral detection of PDs. Multispectral pulses were experimentally obtained for three typical types of PDs in gas-insulated systems. Classification algorithms were adopted to evaluate the three typical types, obtaining success ratios greater than 91%.

In [17], a room-temperature optical humidity sensor based on Nafion, a hydrophilic polymer, is theoretically and experimentally investigated. Results of the relative humidity retrieval with the regression of the physical model parameters are compared to those obtained with different machine learning (ML) techniques.

Convolutional neural networks (CNNs) have revolutionized the ability of computers to analyze and interpret images in a wide variety of applications, ranging from object detection in autonomous vehicles to classifying skin cancer images (for further information, see [18]). These networks, inspired by the visual organization of the human brain, have proven to be highly effective in image classification thanks to their ability to learn and represent the features present in the input data in a hierarchical and discriminative way.

CNNs have become a foundational technology in fields such as computer vision, artificial intelligence, and deep learning. The ability of these networks to automatically learn relevant features from images has greatly simplified the classification process, reducing the need for manual feature extraction.

The references provided below give an overview of recent advances in the field of CNNs and deep learning applied to image classification.

E-cientNet architecture is proposed in [19], which achieves optimal performance in image classification by efficiently optimizing the model size. The contribution of [20] is relevant to image segmentation and has been widely used in object classification applications.

A popular architecture for real-time object detection is studied in [21], and the improved version, YOLOv3, used in image classification applications. The work of [22] focuses on image translation and domain adaptation, which is relevant for image classification in environments with data from different modalities.

Over the years, the architecture of CNNs has undergone constant refinements, giving rise to cutting-edge models such as AlexNet, VGG, Inception, ResNet and other variants, which have set records in image classification competitions such as the Challenge of ImageNet. In [23], an ImageNet Classification with Deep CNNs is studied.

The particular uses of CNNs and signal processing techniques related to PD detection in the field of electrical engineering are summarized below. In [24], the use of CNNs is proposed for PD detection in high-voltage transformers using UHF sensors. In [25], the focus is on the use of CNNs to recognize PD patterns in gas-insulated substations. In [26], an analysis of recent advances in PD classifications using CNNs is made. In [27], PD pattern recognition in GIS-type switches using CNNs with complex data sources is analyzed.

In [28], the CNN model studies PDs, highlighting the importance of not ignoring weak PD signals and the need for extended measurements to improve their detection. This study advances the understanding of epoxy material behavior and the refinement of breakdown detection techniques for practical applications.

A pulse sequence analysis (PSA) method that uses a CNN to recognize defects in power cable splices is presented in [29]. PSA showed effective results compared to conventional methods. The accuracy of the PSA-based CNN model was 95.3%.

In [30], a wireless sensor network (WSN) is combined with a CNN, forming a hybrid framework to detect the pollution status in high-voltage insulators. The WSN comprises the set of sensor readings from each high-voltage insulator on the transmission tower. The sensor readings collected from the sensor network are sent to the processing or sensing unit, where the CNN is used to detect high-voltage insulators with PDs.

Ref. [31] presents a state-of-the-art review on ML-based intelligent diagnostics that have been applied for PD detection, localization, and recognition. Furthermore, technical obstacles are identified that prevent smart PD diagnostics from being applied to the industry, such as insufficient/imbalanced data sets, data inconsistency, and difficulties in cost-effective real-time implementation.

In [32], a PD test is performed on five types of artificial defects in ethylene propylene rubber cables in an HV laboratory to generate signals containing PD data. A total of 3500 sets of PD transient pulses are also extracted, and then 33 types of PD characteristics are established. With these data, a CNN is applied. The typical CNN architecture and key factors affecting the accuracy of CNN-based pattern recognition are described. In [33], a CNN model is proposed to identify and classify different types of events, including internal PD, corona PD, surface PD, and noise.

To our knowledge, the use of these CNN techniques has not been applied in PD analyses in transformer oils with low-cost optical sensors. In this paper, an exhaustive study of the PDs that originate from the bubbles present in the dielectric mineral oil is carried out. These discharges are precursors to electric arc breakdown, and as such represent a method of diagnosing the condition of the mineral oil before breakdown occurs.

To address this problem, the implementation of a combined system is proposed that integrates a high-resolution, low-cost CMOS camera, together with a standard electrical detection system based on a discharge capacitor. The Raspberry Pi 4/2 GB platform is used in conjunction with camera lenses, providing accessibility and portability. The Raspberry Pi Foundation offers computational and digital tools, such as intuitive documentation and interactive development environments for the Python programming language, facilitating the acquisition and subsequent analysis of data.

In the present article, an image classification model is developed using deep learning with a CNN based on a TensorFlow [34] and Keras [35] model. These images are classified into four classes: containing PDs, without PDs, those that present rupture of the electric arc, and finally those that present gaseous bubbles after the rupture of the arc. The effectiveness of the trained CNN is evaluated by validating it with images that were not used during the training process.

This article is divided into the sections listed below. Section 2, Theoretical and experimental background, describes the experimental design of the test device and explains the optical characterization of the measurement system. In Section 3, PD in gaseous bubbles dissolved in oil, the numerical results of the electric potential and electric field intensity for one and two bubbles in a steady state are presented. Section 4, CNN design, training, and validation, describes the design, training, and validation of a CNN using TensorFlow to identify PDs. Finally, in Section 5, Conclusions, the main conclusions are presented.

## 2. Theoretical and Experimental Background

The theoretical and experimental background of the article is summarized below.

Firstly, the experimental design of the test device is presented and the experimental setup used to generate and measure the PDs in the dielectric oil with bubbles is described. It details the main components of the system, such as the power supply, the electrodes, the CMOS image sensor, the discharge capacitor and the polarizers.

Secondly, the optical characterization of the measurement system is carried out and the fundamental optical characteristics of the components used in the experiments are analyzed, such as the compact fluorescent lamp (CFL), the laser, the polarizers and the optical glass of the windows of the measurement cell. Additionally, it explains how polarizers are adjusted to minimize transmitted light and how PD is detected using the Kerr effect and the high-quality camera—an HQ camera.

### 2.1. Experimental Design of the Test Device

#### 2.1.1. HV Laboratory Description

The laboratory has two clearly separated rooms: the HV room where the tests are carried out, and the LV room where the low-voltage (LV) control and the measuring equipment are located.

**Figure 1:**
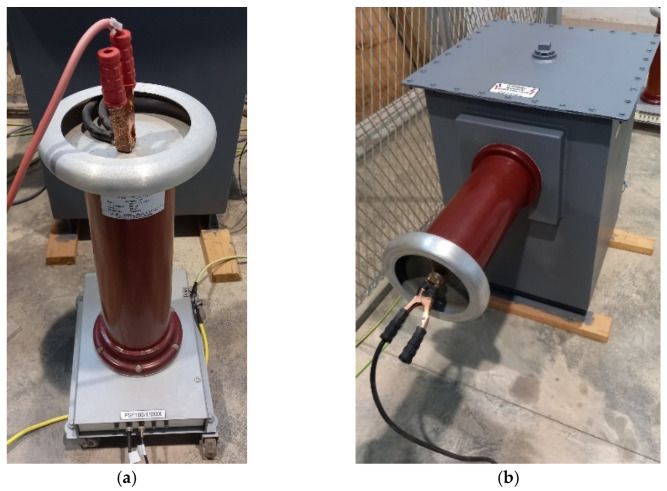
(**a**) Hipotronics discharge capacitor, rated capacitance 1 pF, rated voltage 100 kV, frequency 50 Hz. (**b**) HV test transformer model HHT7-10T-220 (Hipotronics, Hubbell), rated input 230 V AC, 10 KVA, rated output 100 KVA, 100 mA, frequency 50 Hz.

**Figure 2:**
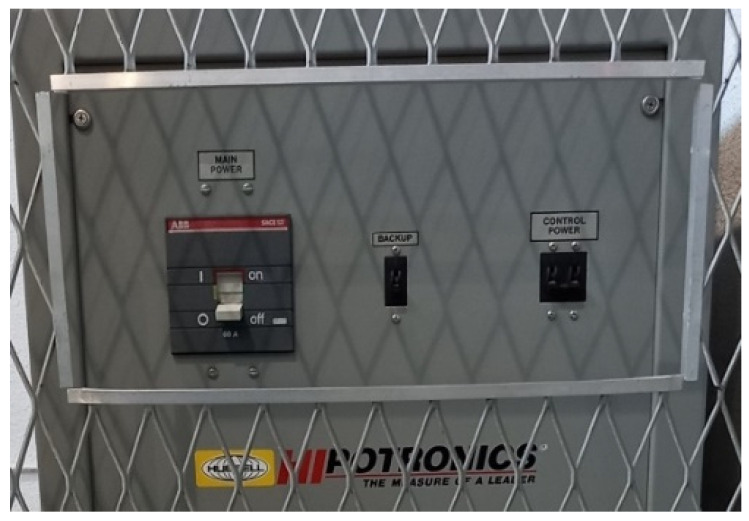
Power supply for the regulation of the output voltage, from 0 to 230 V.

The test equipment, located in the HV room, includes a discharge capacitor (Figure 1a), transformer (Figure 1b), and power supply (Figure 2).

**Figure 3:**
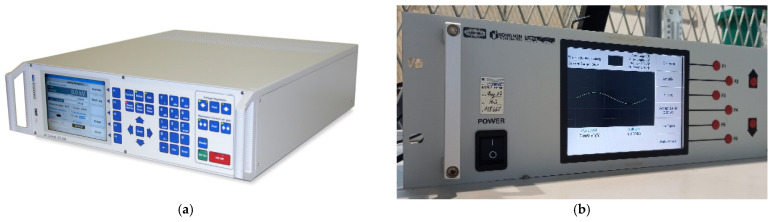
(**a**) Operating terminal OT 248 system, Tettex-Haefely test AG. (**b**) PD detector DDX-9101, Tettex-Haefely test AG.

Located in the control room is the OT 248 system operating terminal control equipment (Figure 3a), which controls the power supply, and the PD detector DDX-9101 (Figure 3b).

**Figure 4:**
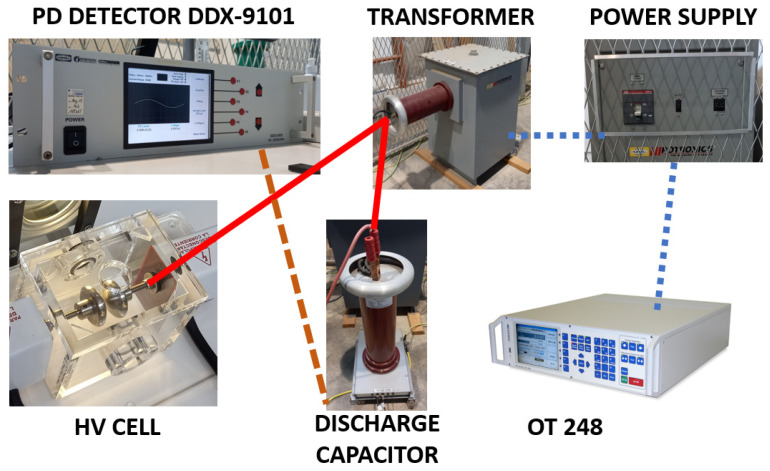
Electrical equipment connection diagram using standard IEC 60270 [36].

**Figure 5:**
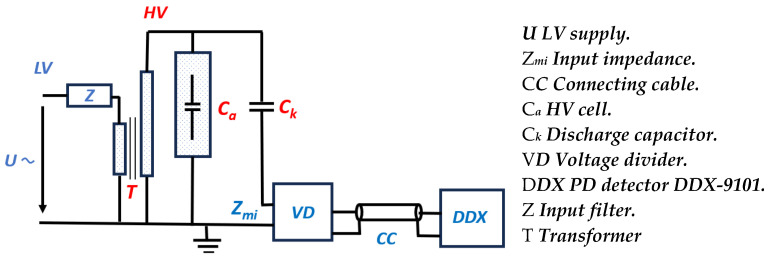
Electrical scheme. Left: electrical scheme using standard IEC60270 [36]. Right: description of electrical scheme.

The OT 248 unit regulates the input LV supplied by the power unit and then supplies the LV input power to the transformer (see Figure 4 and Figure 5). Then, the transformer raises the single-phase voltage to the desired HV. This HV is applied in parallel to the HV cell and the discharge capacitor. The discharge current in the discharge capacitor is measured though the PD detector DDX-9101. Figure 5 shows the electrical scheme following the standard IEC 60270 [36].

#### 2.1.2. Raspberry Pi HQ and V2 Cameras

**Figure 6:**
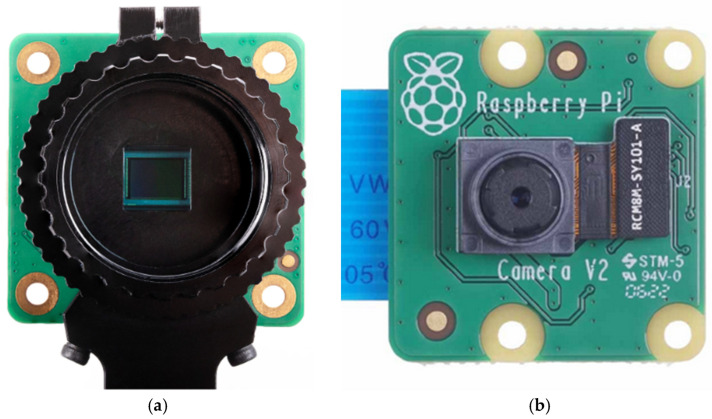
Raspberry Pi cameras: (**a**) HQ (**b**) V2.

Detailed information about the Raspberry Pi HQ camera can be found in [37] (see Figure 6a). It is an affordable camera of exceptional quality with a resolution of 12.3 megapixels and a 7.9 mm diagonal sensor. This camera works especially well in low-light conditions. The M12 mount variant has been designed to be compatible with a wide range of interchangeable M12 lenses. The CS mount variant is optimized for interchangeable lenses in the CS and C-mount formats. It should be noted that for C-mount lenses, the C-CS adapter is included.

**Figure 7:**
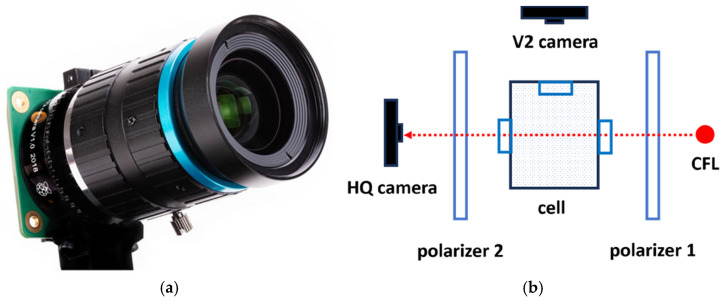
(**a**) 16 mm CGL telelens. (**b**) Location diagram of the HQ and V2 cameras.

In the experiments, the 16 mm CGL telelens with C-CS mount was used, which provides a high-quality image and a low level of distortion (Figure 7a). Figure 7b shows the situation of the HQ and V2 cameras with respect to the polarizers and the CFL.

This camera makes use of the Sony IMX477 sensor [38], an active pixel-stacked CMOS, with a resolution of 12.3 megapixels and a square pixel array with a diagonal of 7.85 mm.

In the laboratory experiments, another camera model, the Raspberry Pi camera V2, was also used (Figure 6b), which makes use of the Sony IMX219 sensor. Table 1 provides a summary comparing the main characteristics of the sensors of both cameras.

#### 2.1.3. Description of the Raspberry Pi 4 Computer

**Figure 8:**
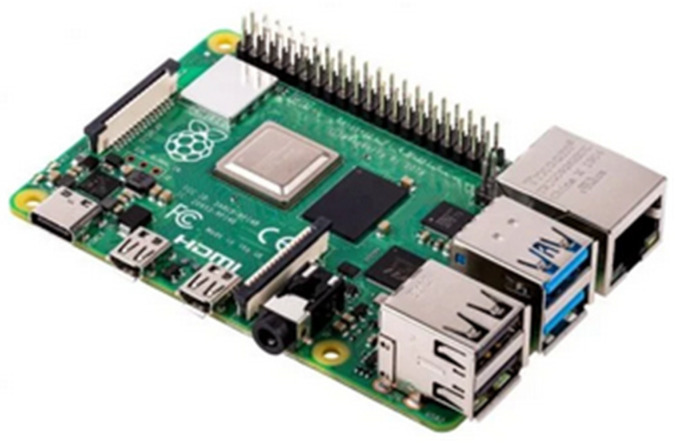
Computer Raspberry Pi 4.

The Raspberry Pi 4 computer [39] (Figure 8) controls the image sensor, collects the images and sends them to the control zone via Wi-Fi. Its main characteristics are summarized below.

#### 2.1.4. Description of the Camera Control Software

The camera modules, HQ and V2, connected to the Raspberry Pi computer offer a wide range of possibilities thanks to the system’s programmability. These applications provide a multitude of options, and their operation can be further simplified using scripting software. The software version used is Python 3.8.

We used the cameras in the experiments with the Raspberry Pi camera libraries that are available in Python to develop custom applications with their features [40].

The practical implementation of the camera modules follows a sequence of four main steps: composition, exposure selection, image or video capture, and post-processing. Composition and focus are initially achieved through a real-time preview window. Exposure time is precisely set to ensure consistent images.

### 2.2. Optical Characterization of the Measurement System

This section details the key optical characteristics of the components used in the experiments, including the CFL, red laser, polarizers, and the optical glass of the windows of the measurement cell.

Firstly, in Section 2.2.1, a spectrum analysis of both the CFL and red laser is carried out. A detailed explanation on adjusting the polarizers is provided in Section 2.2.2. Finally, in Section 2.2.3, PD detection using the Kerr effect and the use of the HQ camera is discussed.

#### 2.2.1. CFL and Laser Light Spectrum Analysis

The light beam from the CFL and the red laser pass through the polarizers and the two quartz glass windows of the measurement cell. We used JGS2 optical quartz glass [41] for the windows.

Quartz glass is recognized for its outstanding performance in the transmission of ultraviolet (UV) light, with extremely low absorption of visible light and near-infrared light in the range of 220–2500 nm, without presenting significant absorption bands. Furthermore, it is characterized by an exceptionally low coefficient of thermal expansion and good chemical stability similar to that of standard optical glass. In summary, quartz glass is considered the optimal material for harsh environments and is classified as UV optical quartz glass with the JGS2 designation.

**Figure 9:**
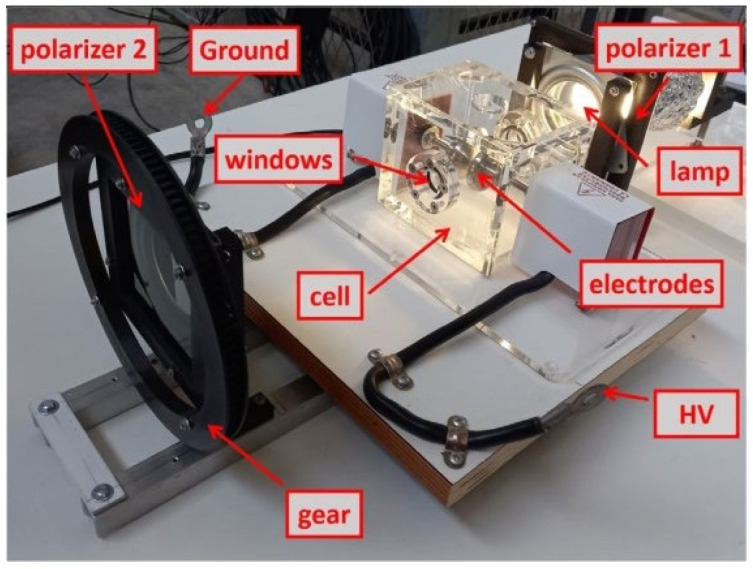
Test device in the HV laboratory.

To achieve precise alignment of polarizers 1 and 2, as clearly shown in Figure 9, a CFL and a red laser were employed.

**Figure 10:**
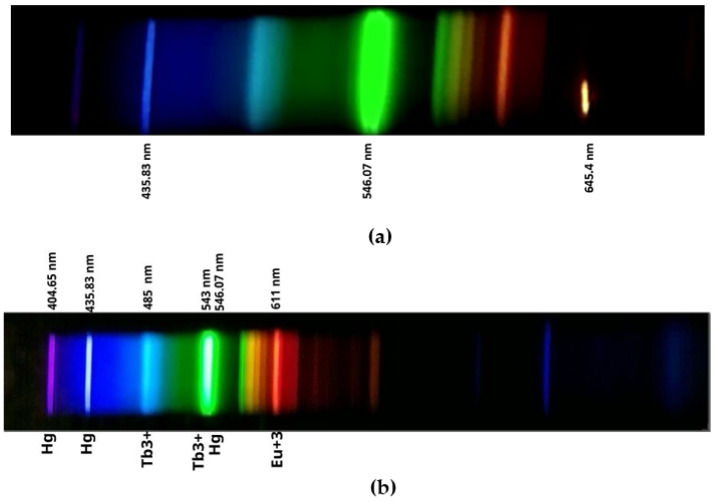
Light spectrum obtained by the V2 camera without infrared filter used to position the polarizers. (**a**) Spectrum of the red laser and CFL used. (**b**) Spectrum of the CFL used, in addition, for the detection of PD with the HQ camera.

We measured the wavelength of the red laser used for the optimal configuration of the polarizers, which was 645.4 nm. The manufacturer specifies it in a range of 650 ± 10 nm. This value and its relationship with the laser spectrum are detailed in Figure 10a.

Additionally, the spectrum of the CFL was realized. It includes lines of 435.83 nm in the violet-blue range and 546.07 nm, in deep green, which are attributable to the mercury present in all CFLs. Additional blue lines can also be observed in the final region of the spectrum, which correspond to the infrared region. This spectrum is represented in Figure 10b. A spectrometer was used with a dispersion coefficient of 0.53 nm/pixel for all measurements carried out in the experiments. The spectrometer images were taken with the V2 camera, without an infrared filter and with the Sony IMX219 CMOS sensor.

#### 2.2.2. Optimal Position of the Polarizers

PDs induce an increase in luminosity in the dielectric oil contained between the electrodes of an HV measurement cell due to the Kerr effect [42]. This increase in luminosity is indicative of the electric field intensity in the PD.

**Figure 11:**
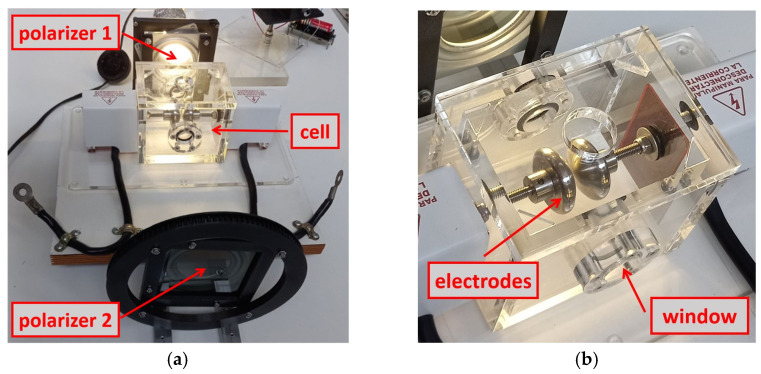
(**a**) Detail of the two square polarizers, the test cell and the one gear. (**b**) Transparent methacrylate cell with the two electrodes.

To improve the sensitivity and contrast in the measurements taken, the light transmitted by the light beam of the CFL and the red laser, optically characterized in the previous section, was minimized. Two independent studies were carried out for each type of lighting. This beam of light, in both cases, passes through the polarizers, parallel to each other, rotated relative to each other at a certain angle that minimizes the passage of light between them (see Figure 11a).

The cell (Figure 11b) is a transparent methacrylate prism that contains two electrodes, one connected to ground and the other to HV. The PD test circuit was set up according to the IEC 60270 standard [36]. The International Electrotechnical Commission (IEC) is a worldwide organization for standardization.

The sides of the cell have two circular windows of quartz optical glass. These allow the light beam to pass, without distortion, between the two electrodes. The upper part of the part is a removable cover that serves to introduce the dielectric oil that surrounds and covers the electrodes. In addition, the upper cover has a circular opening through which a thermometer can be inserted to take measurements of the oil temperature before and after each test. Likewise, it is suitable for taking overhead images of the electrodes during HV tests.

The two polarizers are in parallel planes. One is fixed, polarizer 1, and the other can rotate around the axis perpendicular to its plane, polarizer 2. The movement is produced in a controlled manner thanks to a stepper motor that transmits its rotation through two gears (see Figure 9). The transmission gear, attached to the motor, transmits the movement through a driving belt to a second, larger gear, integral with the mobile polarizer.

**Figure 12:**
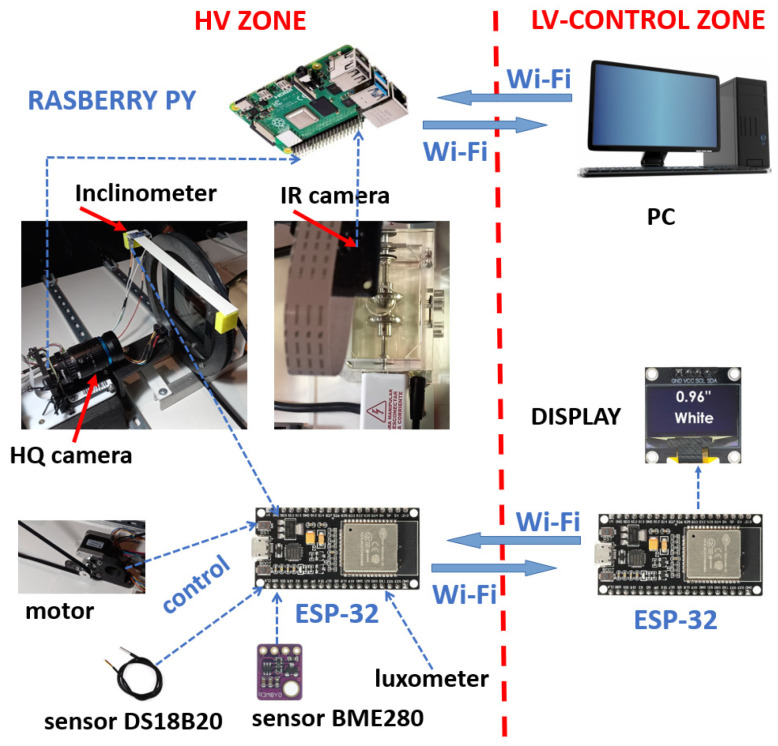
Sensors, actuators, and control system.

An ESP-32 microcontroller controls the slow rotation of polarizer 2. The ESP-32 microcontroller, working as a transmitter, is in the HV room (see Figure 12). It transmits the measured data in real time to the other paired ESP-32 microcontroller, working as a receiver, located in the control room. The two ESP-32 devices use Wi-Fi technology. These data are then presented in a digital display. The transmitter and receiver boards are type ESP-32, 38 pin, dual core, ultralow power, and Wi-Fi and Bluetooth compatible (see Figure 12). The ESP-32s are powered by AC rechargeable 5V DC batteries.

To avoid light pollution caused by natural light or possible laboratory spotlights, a dark room large enough to house the experimental devices and two people, who can intervene in the equipment when not working in HV, was constructed. The approximate dimensions of the dark room are 3 × 3 m in plan and 2.25 m in height. Using a lux meter, it was verified that the luminosity was zero when the interior lights of the chamber were turned off.

We precisely determined the relative position in which polarizer 2 transmits a minimum luminosity, recording the images of a HQ camera and the relative angle of rotation of polarizer 2, using an ADXL345 inclinometer (see Figure 12).

The digital sensor ADXL345 is a small, thin, ultralow-power, 3-axis accelerometer with high-resolution (13-bit) measurement up to ±16 g. Digital output data are formatted as 16-bit two’s complement and are accessible through either a SPI (3- or 4-wire) or I 2 C digital interface. It enables measurement of inclination changes less than 1.0°.

Images acquired by the HQ camera are transmitted by Wi-Fi from a Raspberry Pi transmitter, located in the HV room, to a Raspberry Pi receiver located in the LV room, where a PC displays the images when the experiment is concluded (see Figure 12).

The gray scale is an indirect measure of the intensity of light that goes through the polarizer 2 to the HQ camera. In a grayscale image, each pixel has a value between 0 and 255, where zero corresponds to black and 255 corresponds to white. The values between 0 and 255 are varying shades of gray, where values closer to 0 are darker and values closer to 255 are lighter.

The gray scale was determined based on the relative position of the two polarizers, illuminated successively with a CFL and a red laser using Fiji ImageJ software version 1.54f [43]. Fiji ImageJ is an image processing package, bundling many plugins that facilitate scientific image analysis.

**Figure 13:**
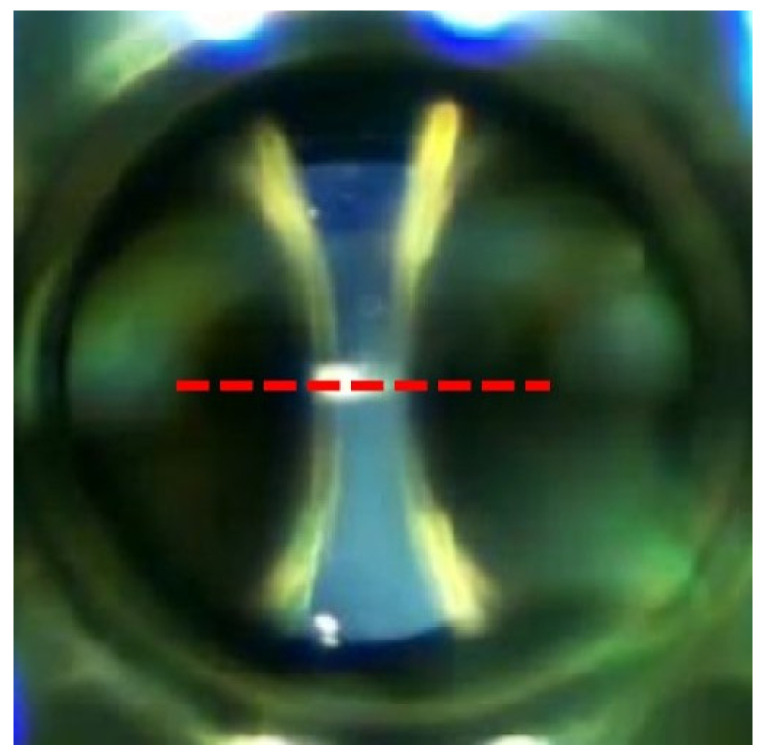
Axial section of the electrodes in which the gray scale was analyzed with Fiji ImageJ.

Figure 13 presents the two electrodes, seen through the circular quartz optical glass window, and the line where the grayscale experimental measurements were taken.

**Figure 14:**
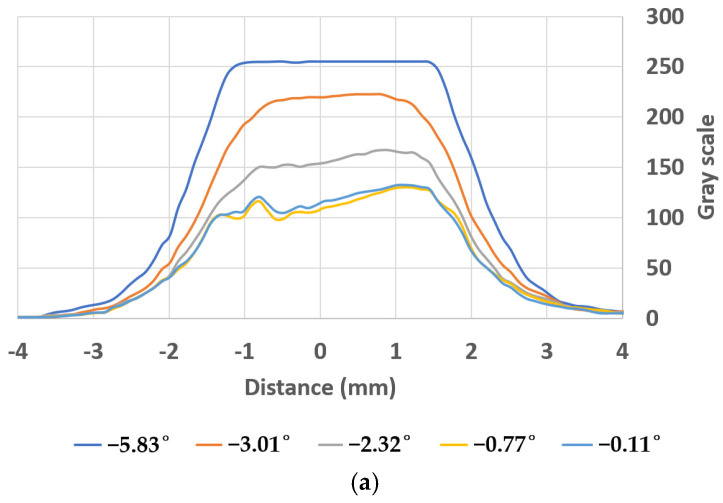

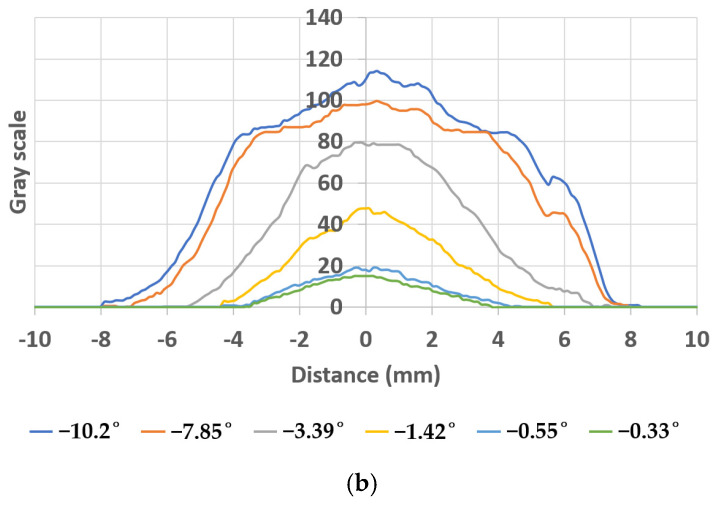
Gray scale for different relative angles of the polarizers. (**a**) For a CFL. (**b**) For a red laser.

The graphs in Figure 14a,b represent the results obtained, that is, they provide the gray scale vs. the relative position in degrees of the two light polarizers. Both present maximums for the central area located between the two electrodes, since in this area the electrodes do not hinder the passage of light.

**Figure 15:**
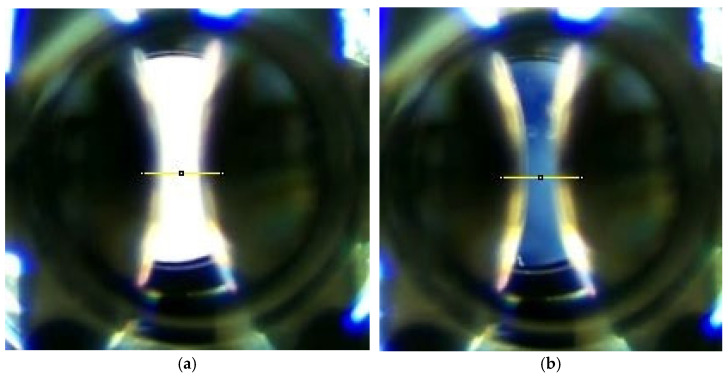
(**a**) Luminosity between the electrodes with the polarizers at −5.83°. (**b**) Luminosity be-tween the electrodes with the polarizers at −0.11°.

Figure 15a,b shows the luminosity for a polarizer position of −5.83° and −0.11°. It can be observed how the luminosity between the electrodes is noticeably lower when the relative position between the polarizers is optimal, close to zero degrees.

The CFL presents a minimum in the gray scale for a phase shift of −0.11° and the red laser beam gives a minimum for −0.33°. We can affirm that the relative angle between the polarizers is optimal, since it is difficult to register a difference of the order of a tenth of a degree. Therefore, the optimal relative position has a phase shift of zero degrees (see Figure 14).

The room temperature, atmospheric pressure, and relative humidity were continuously monitored through the BME280, from Bosch company, manufactured in Germany, 3.3 V digital sensor (see Figure 12). The average room temperature recorded during the experimental tests was 25 °C, the atmospheric pressure 985 mb, and the relative humidity 58%.

A DS18B20 waterproof temperature sensor was used [44] to measure the temperature of the oil before and after the experiments. Temperature changes less than one degree Celsius were detected. Its unique one-wire interface facilitated communication with the devices. It does not require an external power supply unit, as it is powered from the data line. Its stainless-steel probe head makes it suitable for any wet or harsh environment. This thermal sensor was powered from a 3.2 V DC power supply.

#### 2.2.3. PD Detection Using Kerr Effect and HQ Camera

In this section, we begin by defining the magnitudes associated with the Kerr effect. The refractive index, *n*0, according to [45], is defined as the ratio between the speed of light in a vacuum, *c*, and the speed of light in a specific medium, *v*. In our case, when using dielectric oil, this refractive index is calculated as $n_{0}=\sqrt{\varepsilon_{roil}{\cdot \mu }_{roil}}$, which gives us a value of $n_{0}=\sqrt{2.14}$. It is essential to note that in the absence of an electric field, mineral oil exhibits isotropy and therefore has this refractive index.

However, when an electric field *E* is applied to the oil, a double-refraction phenomenon is observed. Along with the refractive index, $n_{0}$, an additional ray appears called the extraordinary ray, which has a refractive index of $n_{e}$. These two indices are related through the well-known Kerr’s law, represented as $n_{e}-n_{0}=kE^{2}$.

Birefringence in oil, which is the double refraction caused by the intensity of the external electric field, is quantified as the difference between these refractive indices, $n_{e}-n_{0}$. The constant *k* is usually referenced for a wavelength of 546 nm. It corresponds to intense green (see Figure 10b). It is obtained by multiplying this wavelength by the Kerr constant, which for a dielectric mineral oil is 1.8·10^−15^ m/V^2^ [45]. This gives us a value of *k* = 9.82·10^−22^ m^2^/V^2^.

Since polarized rays have different refractive indices, their speeds are different. The extraordinary ray, with a higher refractive index, is slower, while the ordinary ray, with a lower refractive index, is faster. The ordinary ray follows Snell’s law of refraction. Furthermore, these two rays are polarized perpendicularly to each other.

When polarizers 1 and 2 (Figure 9) are orthogonal to each other, as explained in the previous subsection, and in the absence of an external electric field, the light, coming from the CFL and reaching the camera, shows a minimum intensity corresponding to ordinary lightning. However, upon applying an electric field strong enough to generate a PD, the intensity of the electric field increases in the region of the PD. This, in accordance with Kerr’s law, causes the extraordinary ray, polarized perpendicularly to the ordinary ray, to pass through polarizer 2 with greater intensity, allowing it to be detected by the HQ camera.

**Figure 16:**
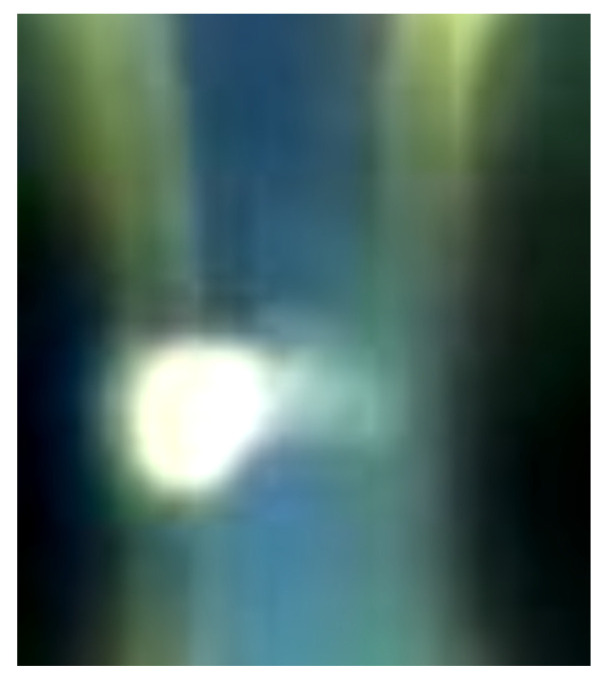
The PD between the electrodes generates an intense electric field concentrated in the discharge area. As a result, the extraordinary ray, which is polarized perpendicularly to the ordinary ray, passes through polarizer 2 with a significantly higher intensity, allowing it to be detected by the HQ camera.

This physical principle is used to visualize the areas of greatest electric field that are generated at the electrodes in the PD regions within the oil, as illustrated in Figure 16.

## 3. PD in Gaseous Bubbles Dissolved in Oil

### 3.1. Validation of the Permanent Regime of the Electric Field of a Bubble

Next, the main PD models and their numerical resolution, in a steady state, for the test cell (see Figure 11b) are explained. In [46], the simulation of PD events in a void space within an insulating material is discussed and several models developed to simulate these PD events. They can be classified into three categories: the induced charge concept model, the three-capacitor model, and the finite element method model, which is a distributed parameter model. Each has its own advantages and disadvantages.

In this work, the distributed parameter model was followed [47], which is more accurate than the other two, because unlike them, it considers the 3D geometry of the measurement cell and the electrodes.

In [47], a distributed parameter model is developed, applying the finite formulation [48] and using effective property parameters. This is the model followed in this work. The main equations are presented below:

(1) $$\tilde{D}M_{\sigma }G\varphi +\frac{\partial }{\partial t}\tilde{D}M_{\varepsilon }G\varphi =0,$$

(2) $$\tilde{D}M_{\sigma }G\bar{\varphi }+j\omega \tilde{D}M_{\varepsilon }G\bar{\varphi }=0,$$

(3) $${\bar{I}}_{t}=-I_{c\;}\left(M_{\sigma }G+j\omega M_{\varepsilon }G\right)\bar{\varphi ,}$$

The most relevant effective physical quantities involved in these equations, which were used in the simulations, are detailed in 47] and can be summarized as follows: the frequency, *f* = 50 Hz, the relative permittivity, $\varepsilon_{roil}$ = 2.14, and the effective electrical conductivity of the oil, σ = 45·10^−12^ S/m, for an oil temperature of *T* = 25 °C.

The boundary condition of the HV electrode is made to match the experimental measurements recorded by the DDX-9101 PD detector. All simulations were carried out in 3D with the Gmsh mesh generator [49]. Although in some cases, the problem has axial symmetry, the situation of the bubbles makes a general analysis in 3D necessary. In these latter conditions, the mesh used has a number of nodes equal to 82,655, the number of triangles is 43,290 and the number of tetrahedra is 441,057. The system of equations set out in Equation (2) is formulated according to the finite formulation. In that case, the solution is equivalent to applying the finite element method. For this reason, the Getdp program [50] was used, which is a general environment for the treatment of discrete problems.

In order to validate the numerical results obtained from Equation (2), they were compared with the results obtained through a known analytical expression [5] that describes the behavior of an electric field in a gaseous bubble. This equation governs the distribution of electric field intensity both inside and outside a spherical bubble at specific points, the poles and the equator.

**Figure 17:**
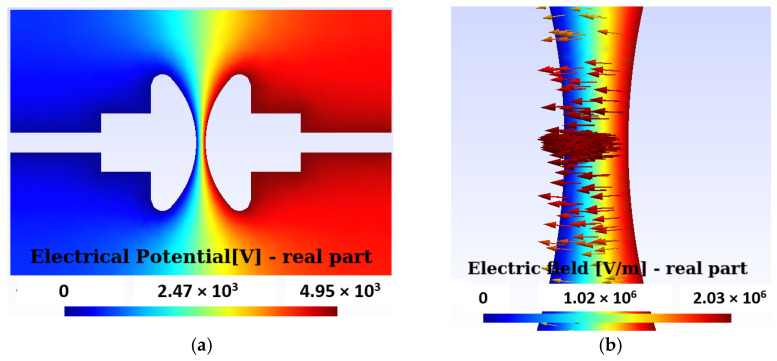
(**a**) Potential distribution between the electrodes for an effective voltage of 3.5 kV. (**b**) The maximum modulus of the electric field is 2.03·10^6^ V/m. The distribution is uniform between the electrodes in their narrowest part without bubbles.

We will begin by presenting the numerical results derived from Equation (2). Figure 17a shows the numerical results corresponding to the electric potential in the absence of breaks and without the presence of a bubble. The potential distribution between the electrodes reflects an effective voltage difference of 3.5 kV. In Figure 17b, the distribution of the electric field intensity can be observed, where the maximum value recorded is 2.03·10^6^ V/m. This distribution is uniform between the electrodes in their narrowest part without the presence of a bubble.

**Figure 18:**
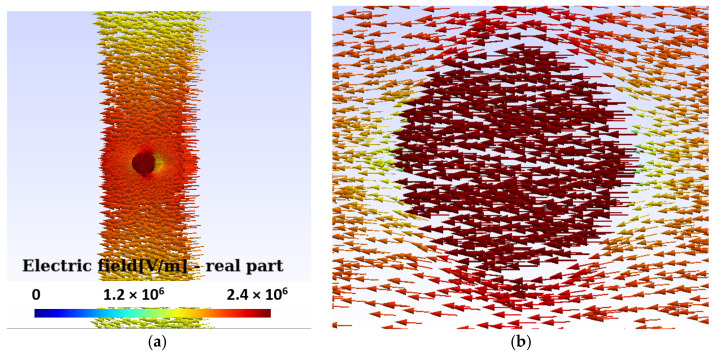
(**a**) The modulus of the electric field intensity is maximum and uniform within the bubble. The distribution of the electric field in the figure is prior to the breakdown of the PD. (**b**) Detail of the increase in the field inside the bubble and its equator, with a decrease in it at the poles.

Then, the distribution of the electric field intensity is presented, both inside and outside, of a bubble located between the two electrodes, obtained by solving Equations (2). Figure 18a shows the modulus of the electric field intensity, which is maximum and uniform within the bubble before a rupture occurs in the PD. Figure 18b provides a detail of the electric field intensity in the vicinity of the bubble.

To corroborate the numerical results obtained in this work, a comparison was carried out with a widely recognized analytical equation. In this case, we consider a gaseous bubble with radius R immersed in a dielectric oil characterized by a relative permittivity constant εroil=εε0. The bubble is in a uniform external electric field, ${\vec{E}}_{0}$, as depicted in Figure 17b. ${\vec{E}}_{0}$, without the bubble, is aligned with the axis of the electrode poles, as shown in Figure 17a. By introducing a gaseous bubble between the electrodes, the electric field $\vec{E}$, inside the bubble, becomes parallel and uniform, as seen in Figure 18. There is an analytical equation [5] that establishes the relationship between the field inside the bubble and ${\vec{E}}_{0}$, as presented in Equation (4):

(4) $$\vec{E}=\frac{3\varepsilon_{roil}}{1+2\varepsilon_{roil}}{\vec{E}}_{0.}$$

At the poles, $\theta =\left[0,\pi \right]$, the electric field intensity of the spherical bubble is less than ${\vec{E}}_{0}$, as described in Equation (5):

(5) $${\vec{E}}_{\theta =\left[0,\pi \right]}=1+\frac{2\left(1-\varepsilon_{roil}\right)}{1+2\varepsilon_{roil}}{\vec{E}}_{0}.$$

It was also confirmed that at the equator, θ=π2, the electric field intensity at the surface of the gaseous bubble increases compared to ${\vec{E}}_{0}$ in the absence of bubble, as illustrated in Figure 18.

(6) E→θ=π2=1+1−εroil1+2εroilE→0.

The quotient in the center of the bubble of the values obtained numerically is EcenterE0=1.182. The value obtained with the analytical Equation (4) is 1.216. It is an error of 2.7%. The electric field intensity distribution in Figure 18a is prior to the breakdown of the PD.

### 3.2. Numerical and Experimental Results with Two-Bubble Rupture

In the previous section, we looked at the distribution of electric field strength, both inside and around a gaseous bubble. This analysis was carried out by comparing the numerical solution derived from Equation (2) with the analytical formulation presented in Equation (4).

**Figure 19:**
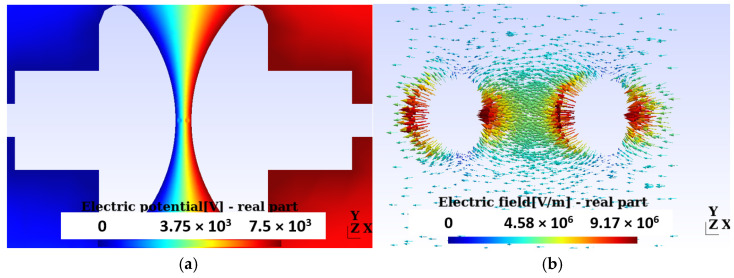
Permanent break condition results. (**a**) Electric potential distribution for an HV electrode boundary condition equal to 5.3 kV effective. From this voltage, the first PDs are observed. The two bubbles used in the simulation are observed. (**b**) Electric field distribution around the two bubbles between the electrodes with PD. The electric field intensity is maximum at the poles and minimum at the center of the bubbles.

In this section, we proceed to an evaluation of the distribution of the electric potential and the intensity of the electric field in the area surrounding two bubbles in the region between the electrodes, with the condition of a PD. Figure 19a graphically represents the distribution of the electric potential around two bubbles, each with an approximate diameter of 0.6 mm. This distribution was obtained under a constant boundary condition on the HV electrode, set at 5.3 kV effective.

From this voltage, in the laboratory, the first PDs were observed. The analysis was carried out in a 3D domain because the general arrangement of the bubbles does not allow an axially symmetrical approach.

Figure 19b provides a view of the electric field intensity distribution in the vicinity of the two bubbles located between the electrodes during a PD. It is relevant to highlight that the electric field intensity reaches its maximum value at the poles of the bubbles and gradually decreases until reaching its minimum value at the center of the bubbles.

**Figure 20:**
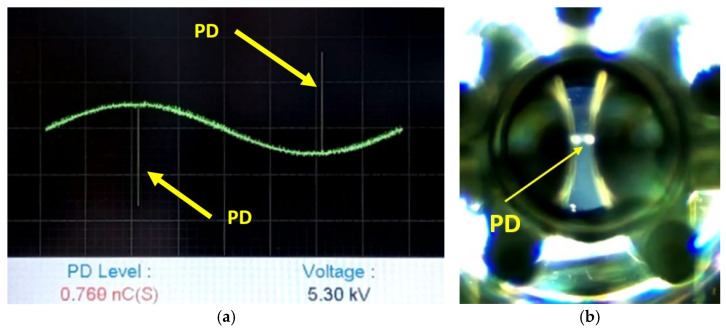
Experimental results for PD using the HQ camera and PD detector DDX-9101 for an effective voltage of 5.3 kV. (**a**) Electrical measurement of the PD observed in Figure 20b. (**b**) Image obtained with the HQ camera corresponding to Figure 20a.

The boundary condition of 5.3 kV used in the simulation was set based on the experimental measurements obtained through the DDX-9101PD detector, with an approximate induced charge of 0.760 nC, as illustrated in Figure 20a. Additionally, in Figure 20b, the image captured by the HQ camera is presented, through an optical glass and specific polarizers, under the same conditions of potential difference of 5.3 kV.

## 4. CNN Design, Training, and Validation

### 4.1. Introduction

In order to carry out the design, training and validation of the CNN, the computer applications TensorFlow and Keras were used. Keras is a Python library for machine learning and deep neural networks. Keras can run on top of TensorFlow. It was created by Google Brain in 2011. Since 2015, it has been open source [18]. In TensorFlow 2.0 and later, Keras has become the default high-level application programming interface (API) for defining and training neural networks within TensorFlow.

TensorFlow has a backend component that is programmed in more efficient and low-level programming languages, such as C or C++. This is important because Python, although a versatile and easy-to-use language, tends to be slower in executing certain mathematical operations and intensive calculations compared to languages like C/C++.

All our simulations with TensorFlow were carried out in the Google Colab environment. Colaboratory or Google Colab is a free online platform. It allows users to run and write code in Python through their web browser. It is based on the Jupyter Notebook development environment. It offers a cloud runtime environment that includes access to powerful computing resources, such as Google’s graphics processing units (GPUs) and tensor processing units (TPUs).

This section details the process that was followed, from the initial collection of images, and their subsequent classification, as described in Section 4.2. Subsequently, the artificial enrichment of the images is addressed by transformations before carrying out the training, which is explained in Section 4.3.

Then, we proceed to the creation and design of the most suitable convolutional neural network, as explained in Section 4.4. Next, the model is compiled. This is a crucial step that is documented in Section 4.5.

The network training process is detailed in Section 4.6, where the specific parameters and settings used in TensorFlow, within the Google Colab environment, are explained in depth to facilitate more precise monitoring. Two fundamental aspects of neural network convergence are addressed in Section 4.7 and Section 4.8, which focus on convergence for three and four classes, respectively, providing a detailed understanding of how the network adapts to different data sets and requirements.

Finally, model validation is carried out, where its performance is tested using images that were used in the CNN training process. This is described comprehensively in Section 4.9.

In each of these subsections, detailed explanations of the parameters and functions used in TensorFlow, in the Google Colab environment, are included with the purpose of offering a complete and precise guide to the entire process carried out in this work.

### 4.2. Image Collection and Classification into Classes

**Figure 21:**
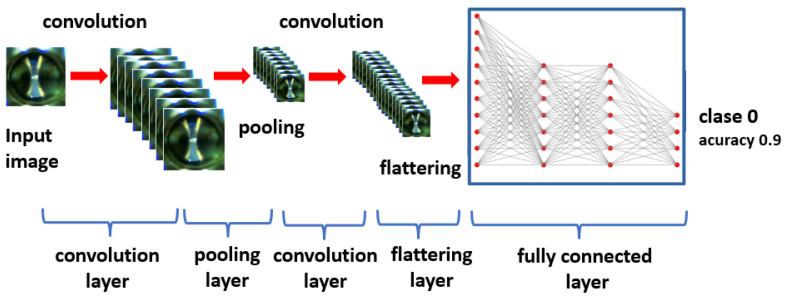
The objective of the CNN is to determine from an image which class it belongs to and to quantify its accuracy.

The main objective of the CNN is to analyze images and determine which category they belong to, in addition to quantifying the accuracy of this classification. This can be seen in detail in Figure 21.

Figure 21 shows a visual representation of the CNN. It presents the following layers:

- Convolutional: in this layer, each filter is applied to the image in successive positions along the image, and through convolution operations, a features map is generated.
- Pooling: the aim of this layer is to reduce the computational load by reducing the size of the feature maps.
- Fully connected: this layer takes the convolutional features, previously flattened, generated by the last convolutional layer and makes a prediction.

To carry out this process, it is essential to have a set of images as broad and diverse as possible, along with their respective classification into classes of interest. In our case, we have a dataset that consists of a total of 2000 images.

**Figure 22:**
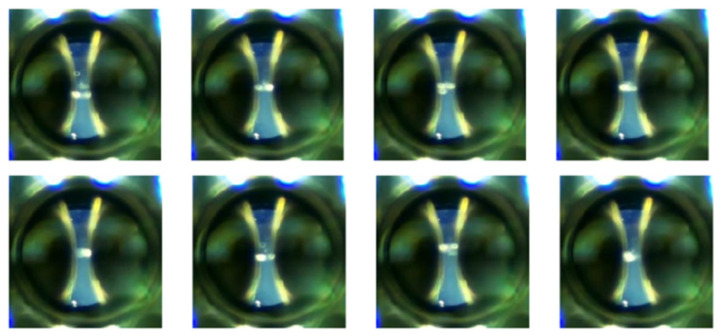
Eight random images out of a total of 500 belonging to class 0 called PD.

**Figure 23:**
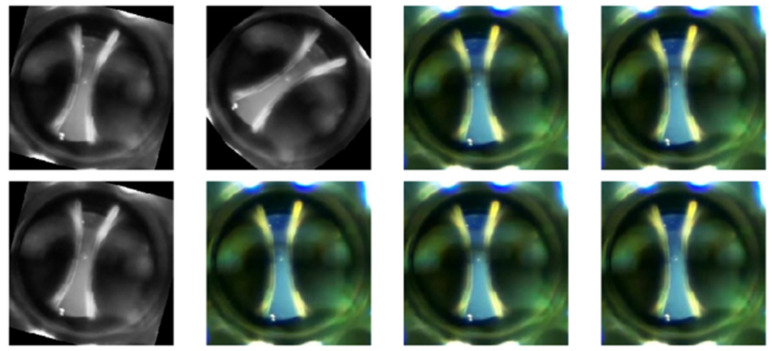
Eight random images out of a total of 500 belonging to class 1 called NO_PD.

**Figure 24:**
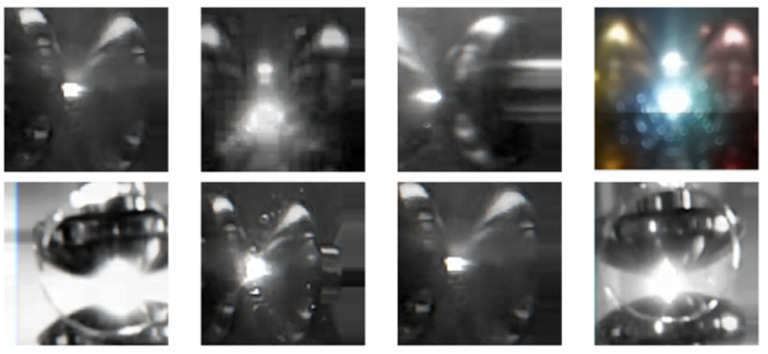
Eight random images out of a total of 500 belonging to class 2 called ARC.

**Figure 25:**
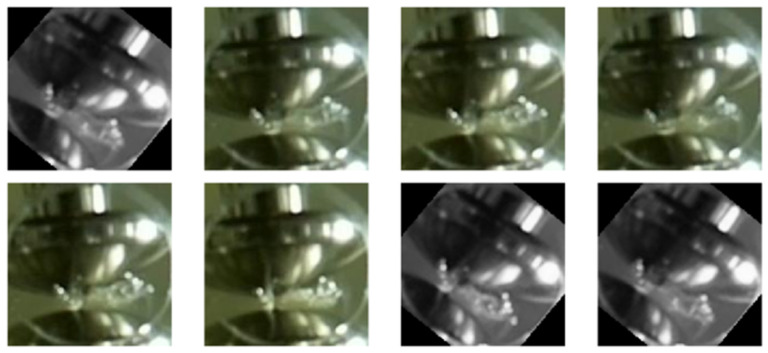
Eight random images out of a total of 500 belonging to class 3 called BREAK.

In our experiments, we defined four specific classes:

- Class 0—PD (with PD): This category is illustrated in Figure 22, where eight images randomly selected from a set of 500 are shown. These images represent different instances of the PD, which were experimentally verified through electrical measurements through the discharge capacitor.
- Class 1—NO_PD (without PD): Figure 23 presents eight random images out of a total of 500 that belong to this category. These images represent moments in which the PD detector DDX-9101 does not detect any PD.
- Class 2—ARC (total rupture of the electric arc): Figure 24 shows images that correspond to the moment of total rupture of the electric arc.
- Class 3—BREAK (post-arc break with a large number of bubbles): Finally, Figure 25 shows a random selection of eight images out of a total of 500 belonging to this class.

In summary, the main objective of the neural network is to classify images into these four categories, and to achieve this, a large and varied dataset consisting of 2000 images in total was used. Each class has its own identification and is supported by a representative sample of images from its respective category.

### 4.3. Image Transformation

In order to strengthen the model’s ability to generalize and make accurate predictions, new images were obtained through the HQ camera. For this, the ImageDataGenerator() function was used. This function makes it possible through transformations to create multiple variations of the original images, thus enriching the data set and improving the generalization of the model. This function was applied before training the CNN model.

Some of the main arguments used in our work by this function are detailed below:

- “rescale = 1/255” is a simple normalization, where the value of each pixel in the image is divided by 255. This is done to ensure that the pixel values are in the range [0, 1], which facilitates processing and improves convergence during training.
- “rotation_range = 30” allows us to randomly rotate images within a range of ±30 degrees. This helps the model to be more robust to different object orientations.
- “width_shift_range = 0.25” and “height_shift_range = 0.25” allows us to shift images horizontally and vertically within a range of ±25% of the original image size. This simulates variations in object position in the image and allows the model to support greater variability in object locations.
- “shear_range = 15” introduces warps that cause the image to be sheared at an angle of ±15 degrees. This is useful for working with images taken from different angles.
- “zoom_range = [0.5, 1.5]” is a transformation that randomly zooms images in the range 0.5 to 1.5. This simulates the variability in the distance from the camera to the object and improves the model’s ability to recognize objects in different sizes.
- “validation_split = 0.2” sets the proportion of data used for validation. In this case, 20% of the data were separated to be used as a validation set, while the remaining 80% was used for training.

Similarly, the “flow_from_directory()” function was employed, which is used in the context of data processing and preparation for training deep learning models. This function simplifies the loading and pre-processing of images stored in a folder structure organized as in the previous section.

Below are described some of the arguments used in this function.

- “/content/drive/MyDrive/Colab_Notebooks/dataset” is the path to the directory containing the training data. In this case, the data are organized into subfolders, where each subfolder represents an object class; in our case “PD,” “NO_PD,” “ARC” and “BREAK.”
- “target_size = (224, 224)” sets the size to which all images will be resized. The images were resized to a size of 224 × 224 pixels, which is a size commonly used in many deep learning applications.
- “batch_size = 32” defines the batch size for training. During training, the images were grouped into batches of 32 images each to optimize the training process.
- “shuffle = True” was set to True so that the images within each batch were randomly shuffled at each training epoch. This helps ensure that the model does not memorize the order of the images.
- “classes = [“PD,” “NO_PD,” “ARC,” “BREAK”]” sets the list of class names to which the images belong. This is essential so that the data generator knows what labels to assign to the images based on the folder structure.

**Figure 26:**
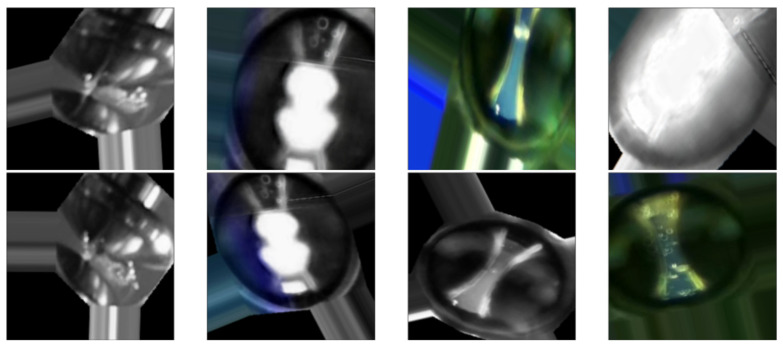
Examples of images transformed by the ImageDataGenerator() function. They improve the generalization of the dataset.

**Figure 27:**
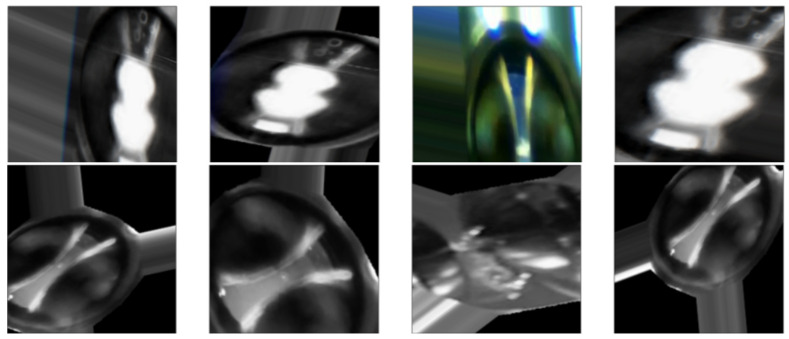
Examples of images generated by the ImageDataGenerator() function. They enrich the images of the dataset.

Figure 26 and Figure 27 show some random examples of images transformed and generated with the ImageDataGenerator function.

### 4.4. Creation of the CNN Model

There are many advantages in adopting a CNN design. First, convolution layers specialize in learning to extract relevant features from images. This is essential for visual information processing. Second, clustering layers play a crucial role in reducing the spatial dimension and the number of parameters in the model. This not only improves computational efficiency but also mitigates the risk of overfitting. Third, the concept of dropout becomes a valuable ally by preventing overfitting through the introduction of a controlled degree of randomness during the training process. Finally, the dense layers at the end of the model play a crucial role in allowing final decisions to be made based on the features extracted throughout the process.

The program responsible for conducting CNN training and validation is Python.

The function “model = tf.keras.Sequential()” was used to create a CNN model in TensorFlow using the Sequential interface, which is a simple way to define a linear sequence of layers in the neural network.

Each of the sets of layers, which correspond to the arguments of the function “model = tf.keras.Sequential()” used in this model, are explained in detail below, as well as the advantages of each one:

- “tf.keras.layers.Conv2D(32, (3,3), input_shape = (224,224,3), activation =”relu”)” is a 2D convolution layer that applies 32 filters, also known as kernels, to the input image. Each filter is 3 × 3 pixels in size.
- “input_shape = (224,224,3)” specifies the size of the input image. The images are 224 × 224 pixels with RGB color channels. The “relu” (rectified linear unit) trigger function is applied after convolution. This function is common in CNN and helps to introduce nonlinearity in the network.
- “tf.keras.layers.MaxPooling2D(2,2)” is a pooling layer that reduces the spatial dimension of the output of the convolution layer. It uses the max-pooling method with a window size of 2 × 2 pixels and retains only the maximum value of those pixels. This method simplifies the number of parameters and operations in the network, which helps reduce the risk of overfitting and improves computational efficiency.
- “tf.keras.layers.Conv2D(64, (3,3), activation = “relu”)” is similar to the first convolution layer, but now 64 filters are used instead of 32.
- “tf.keras.layers.Dropout(0.5)” is a dropout layer that randomly deactivates 50% of the neurons in this layer during training. Dropout is a regularization technique that helps prevent overfitting by reducing interdependence between neurons.
- “tf.keras.layers.Flatten()” converts the output of the previous layer into a one-dimensional vector.

### 4.5. Compilation Stage

Setting up the “model.compile()” function is a critical step in building and training a neural network.

Through this function, several parameters are specified that influence how the model learns and evaluates its performance. The following details how these key parameters were implemented, each with a specific role in configuring the network and optimizing its ability to solve the problem.

- “optimizer = “Adam”,” where the Adam optimizer is an optimization algorithm that automatically adjusts the learning rate during training.
- “loss = “categorical_crossentropy”,” which is the loss function used during training. Our case is a multiclass classification problem, so categorical cross-entropy loss is a common option. It helps measure the difference between the probabilities predicted by the model and the true labels of the data.
- “metrics = “accuracy”” defines the metrics that are used to evaluate the performance of the model during training and evaluation. In this case, the accuracy metric is used, which measures the proportion of samples classified correctly.

### 4.6. CNN Model Training

In TensorFlow, the model.fit() function plays a vital role in training a neural network using training data. Through this function, the model training process is configured and monitored.

The arguments that are provided to the model.fit() function are essential to customize and direct this training process effectively.

Below, we detail how these key arguments were implemented, each with a specific role in configuring the network and optimizing its ability to solve the problem:

- “training_data” represents the set of training data used to train the model. It contains the input features and corresponding output labels for training. It is provided as a pair of tensors: one for features and one for labels.
- “epochs” specifies the number of epochs or training cycles that are performed. A complete epoch occurs when the entire training dataset has been used once to update the model weights. The value that was used in training in this work was “epochs = 50.”
- “batch_size = 32” defines the size of the data batch used in each training step. At each epoch, the training data are divided into batches of the specified size and used to calculate the gradient and update the model weights. A batch size larger than 32 can speed up training but requires more memory.

### 4.7. Convergence for Three Classes

Neural network design involves computationally intensive processes that can consume a significant amount of time. With the purpose of optimizing execution times and determining the optimal method, along with its parameters, the work begins with an initial focus on a set of three specific classes.

These classes are the PD class, which covers situations with PD, the NO_PD class, which refers to instances where PD does not occur, and the BREAK class, which encompasses all images after the breakdown of the electric arc, including all the ARC class. This initial selection of three classes allows for a more agile and precise evaluation of the methods and parameters, thus accelerating the neural network design process.

Three methods were evaluated, which can be implemented using TensorFlow. We wanted to know which of the three was most efficient from a computational point of view. These three methods are called the CNN method with knowledge transfer, the CNN method without dropout, and the CNN method with dropout.

#### 4.7.1. The CNN Method with Knowledge Transfer

**Figure 28:**
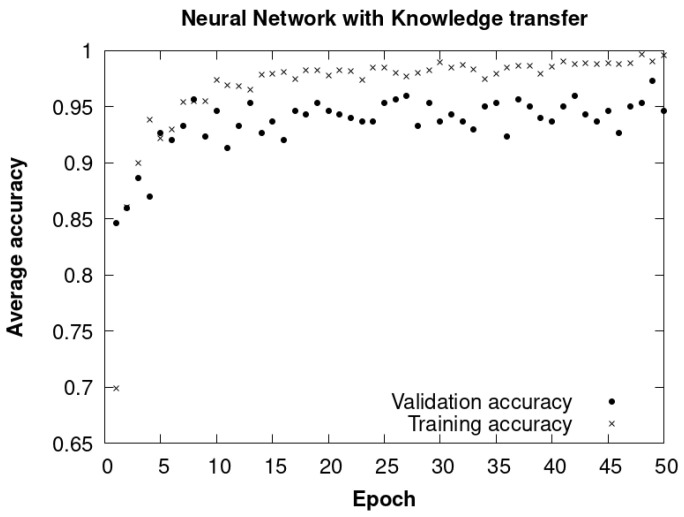
Knowledge transfer method. Average accuracy of training and validation data with epochs.

The results of the knowledge transfer method are seen in Figure 28. They represent the average accuracy of the training and validation data over the epochs. These visualizations were generated using TensorFlow and TensorFlow Hub, leveraging a pretrained model known as MobileNetV2.

MobileNetV2 is a widely recognized CNN model used in computer vision tasks due to its efficiency and high performance. It was used as a starting point to build a new three-class classification model.

It is important to highlight that the weights and internal layers of MobileNetV2 were set as non-updatable during the subsequent training process. This means that the MobileNetV2 model remained unchanged and worked as a static feature extractor, avoiding the need to retrain it. This transfer learning strategy contributed significantly to reducing execution times, achieving a total training duration of only 1.22 h.

To ensure compatibility with MobileNetV2, the expected input data were configured with dimensions of 224 × 224 pixels and RGB color channels. The dataset used for training consisted of a total of 1200 images distributed in three different classes, while the verification set consisted of 300 images equally distributed in the same three classes. The training process was carried out over 50 epochs, and no significant improvements in model performance were observed beyond this point.

We observed that there was a stabilization in accuracy at between 40 and 50 epochs. Therefore, it was decided to carry out all the experiments with this maximum value of epochs for all the cases considered, as seen in Figure 28. Furthermore, from 50 epochs onwards, calculation times increase significantly without obtaining significant improvements in accuracy and with risks of overfitting.

The CNN had a total of 2,261,827 parameters, of which 3843 were trainable and 2,257,984 non-trainable. This information provides a complete overview of the configuration and performance of the model used in the classification process.

#### 4.7.2. CNN Method without Dropout

In this second method implemented in TensorFlow, a model that incorporates convolutional layers interleaved with max-pooling layers was used with the purpose of extracting relevant features from the input images. These features were then flattened before passing through two fully connected dense layers, responsible for carrying out the final classification. It should be noted that the output layer of this model consisted of three units, indicating that the model was designed to perform classification into three different classes.

**Figure 29:**
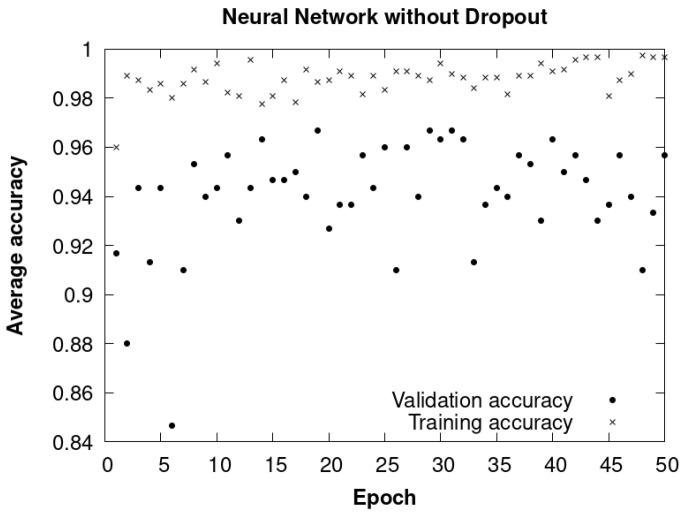
CNN without dropout. Average accuracy of training and validation data throughout epochs.

The results obtained from this second method are represented in Figure 29. It reflects the evolution of the average accuracy during the training and validation process throughout the different epochs.

Regarding the technical characteristics of this model, it is important to highlight that the input images have dimensions of 224 × 224 pixels and three color channels in the RGB space. The execution time, without the inclusion of dropout techniques, was around 2.20 h. The training set consisted of a total of 1200 images distributed in three classes, while the validation set consisted of 300 images, also distributed in the same three classes. This model had a total of 18,682,195 parameters, all of them considered trainable. The training process was carried out over 50 epochs.

Compared to the first method implemented, it is evident that the results obtained in terms of validation accuracy are less favorable in this second approach.

#### 4.7.3. CNN Method with Dropout

In the third method, also implemented in TensorFlow, an additional technique called dropout is introduced, configured using the argument “tf.keras.layers.Dropout(0.5).” Dropout is a regularization strategy widely used in neural networks with the purpose of mitigating overfitting and enriching the generalization capacity of the model.

The application of the dropout, with a value of 0.5, implies that during the training process, approximately half of the neural connections are randomly deactivated in each iteration. This has the effect of preventing the model from becoming overly reliant on specific neural connections and features, which in turn can boost its ability to generalize and adapt to previously unseen data. The training covered 50 epochs, and the total execution time was approximately 2.20 h.

**Figure 30:**
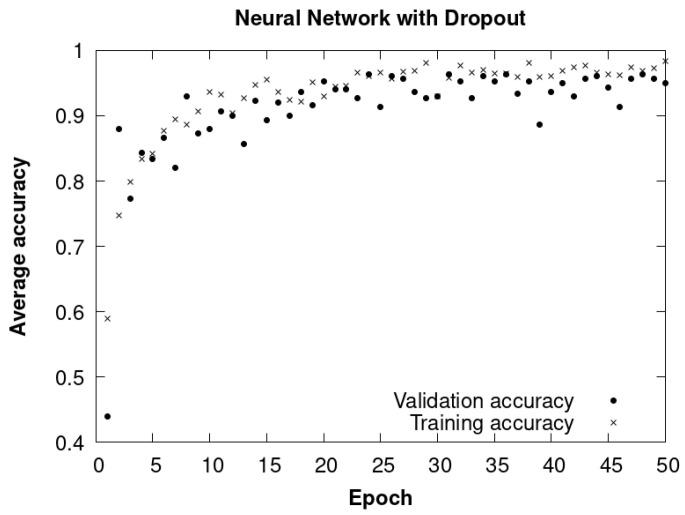
CNN with dropout. Average accuracy of training and validation data throughout epochs.

The results derived from this third method are represented in Figure 30, showing the evolution of the average accuracy during the training and validation stages throughout the various epochs.

By incorporating the dropout technique in this third method (Figure 30), a significant improvement in the accuracy and generalization capacity of the model was achieved compared to the second method that does not have dropout. However, the results are very similar to those obtained with the first method with knowledge transfer.

### 4.8. Convergence for Four Classes

In the previous section, the three CNN methods were compared. In this section, the study of CNN with dropout and with knowledge transfer is carried out for the four classes, as they were found to be the two best methods, achieving similar results.

**Figure 31:**
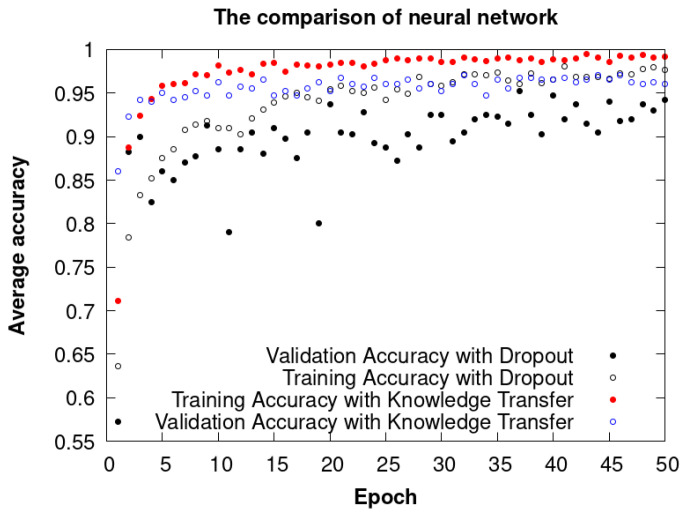
CNN with dropout and transference methods trained with the four classes. Average accuracy of the training and validation data throughout the epochs, with a final average accuracy around 95%.

Figure 31 shows the evolution of the average accuracy throughout the different epochs during the training and validation process. The estimated time necessary to complete the 50 training epochs was approximately three hours for both methods.

A final average accuracy of the training and validation data greater than 95% was obtained, with 50 epochs, as can be seen in Figure 31.

The average accuracy with both CNN dropout and with knowledge transfer is similar to the four classes, as can be seen in Figure 31. They achieved values higher than 95% in fewer than 50 epochs. However, considering that CNN with dropout was not trained previously, we assume that this CNN takes more epochs to reach the same accuracy. As it is possible that a CNN knowledge transfer model may not be accessible, we decided to use CNN with dropout from this point forward.

**Figure 32:**
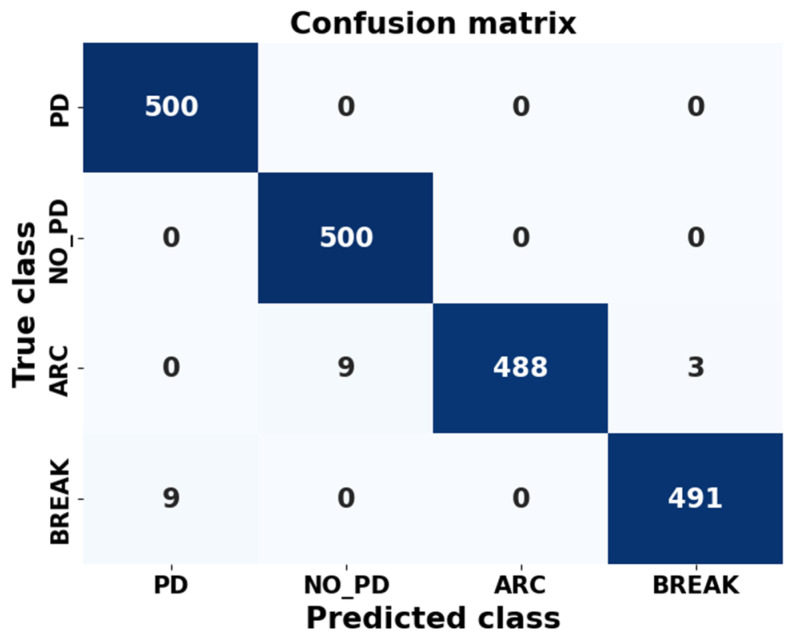
Confusion matrix for the four classes of the dataset.

Once the final model had been obtained, the confusion matrix was generated for the four classes. It is represented in Figure 32.

In this matrix, the true labels are on the *y* axes. They correspond to the actual labels associated with each image in the test dataset. These labels were obtained by loading the test images and assigning a label to each one, based on the directory the image came from. These labels represent the actual class to which each image belongs. The predicted labels, *x* labels, are the labels that the model generated for each image in the test dataset after performing inferences with the pretrained model. These labels represent the class that the model assigns to each image.

The “confusion_matrix” function calculates and returns a square matrix that has dimensions equal to the number of classes in the classification problem. In this matrix, the rows represent the ground truth classes, while the columns represent the classes predicted by the model.

Each element of the confusion matrix in row *i* and column *j* indicates the number of samples that belong to class *i*, according to the true labels, and that were classified as class *j* by the model. In other words, it represents how many times the model was correct in predicting class *j* when the true class was *i*.

**Figure 33:**
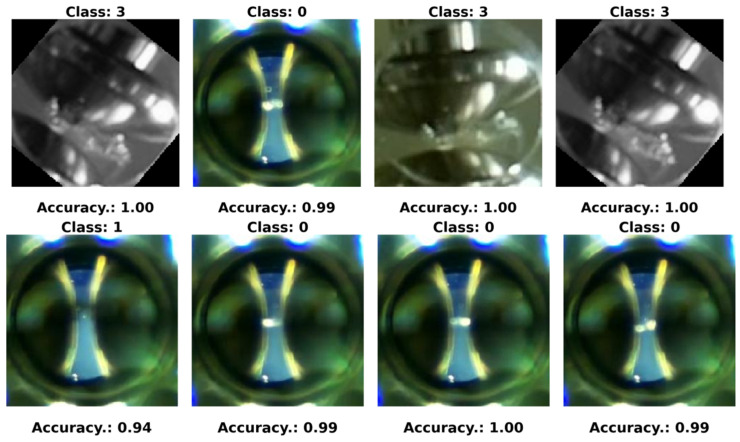
Eight random images obtained from the dataset.

**Figure 34:**
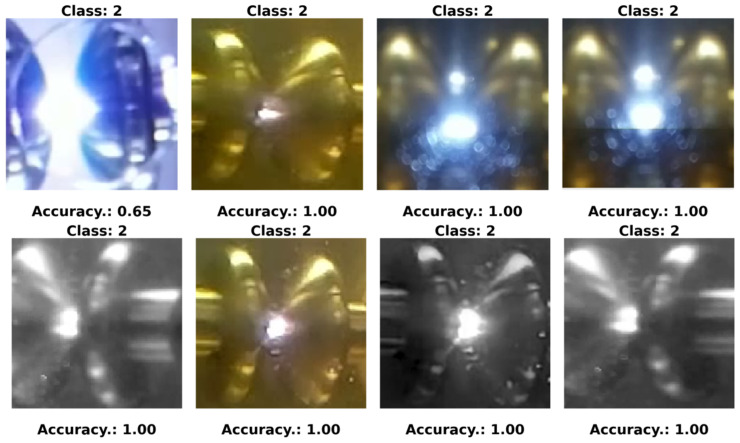
Eight random images obtained from the ARC class set.

**Figure 35:**
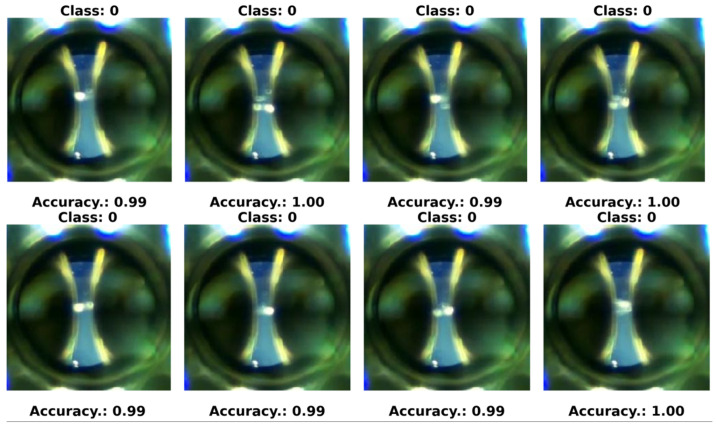
Eight random images obtained from the PD class set.

To graphically visualize the performance of the neural network trained in the last configuration, eight random images are presented in Figure 33, Figure 34 and Figure 35. These images were randomly selected from the dataset, from the ARC class and from the PD class, respectively. Additionally, the predictions made by the neural network for each of these images are included.

### 4.9. CNN Test

In this subsection, we carry out the verification of the neural network previously trained in Section 4.8 by using a test dataset that was not used during the network training process.

Firstly, we selected a total of 72 images corresponding to 72 instances of breaks, class ARC, which came from the experiments that were not used in the training of the neural network. These experiments were carried out with a cell that is described in [15]. This stage focuses on testing the model.

The 72 instances of rupture are divided into three sets of tests, which correspond to test speeds of 3 kV/s, 2 kV/s and. 0.5 kV/s. To provide detailed context on the specific conditions of the experiments and image collection of these arrays, summaries are presented in Table 2, Table 3 and Table 4.

**Figure 36:**
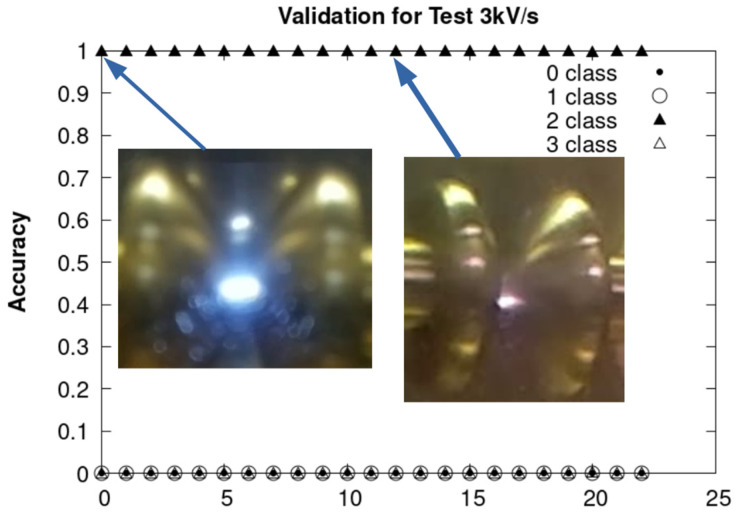
Experiment ramp of 2 kV/s.

**Figure 37:**
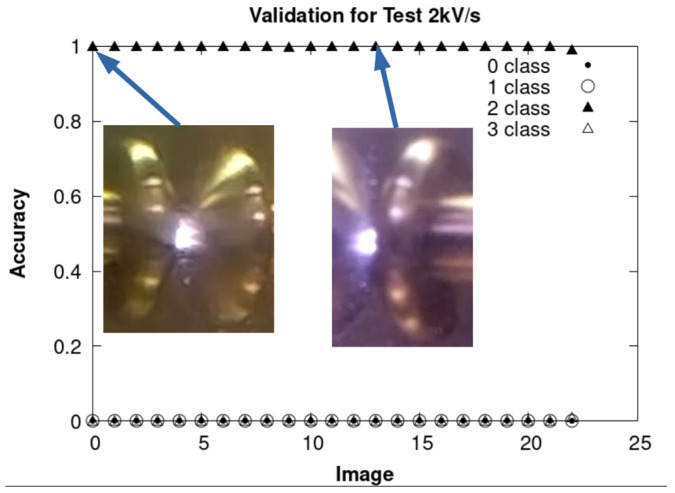
Validation of the ARC class with experiments carried out at 2 kV/s.

**Figure 38:**
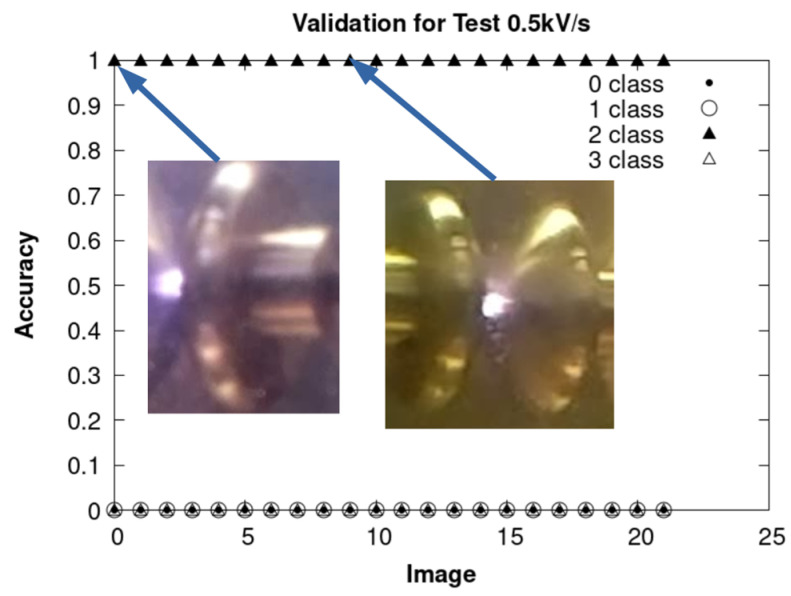
Validation of the ARC class with the experiments carried out at 0.5 kV/s.

The results obtained by using CNN are presented, respectively, in Figure 36, Figure 37 and Figure 38 for each of the sets of tests mentioned above. In the three figures, the accuracy is found to be 100% for the ARC class.

The images were captured with three Raspberry Pi V2 cameras, located on the right, R, in the center, C, and on the left, L, of the cell. Each camera captures different frames per second (fps). The room temperature (T) was measured in all the experiments. The experiment ends when the breakdown voltage V_rup_ is achieved.

**Figure 39:**
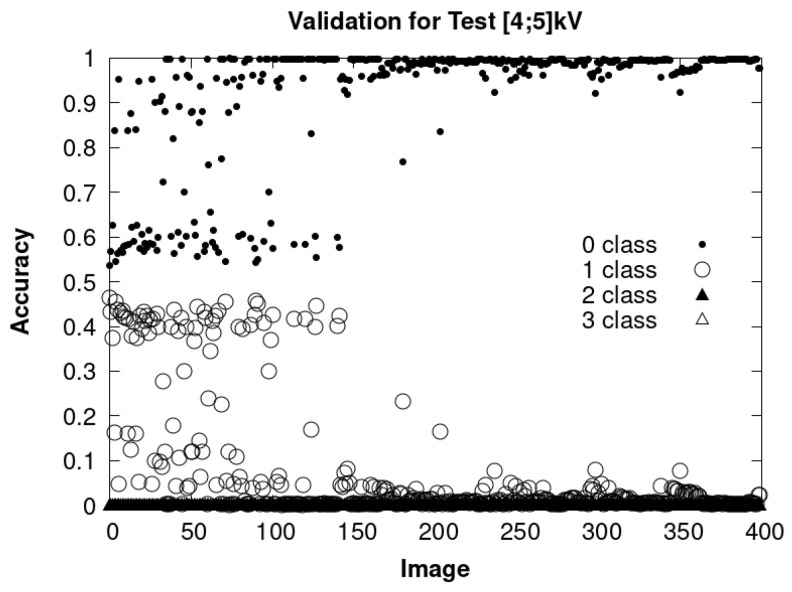
Graph with 400 images obtained at the limit where PDs begin between 4 and 5 kV.

Secondly, in this testing stage, PD images were analyzed in two different sets, composed of 400 and 571 images, captured using the HQ camera. Of the 400 images, these correspond to PD tests for voltages varying between 4 and 5 kV. The results of this analysis are presented in Figure 39. This graph shows an improvement in the identification of the PD from image number 150, which is due to the fact that the voltage is closer to 5 kV where PDs are more frequent.

**Figure 40:**
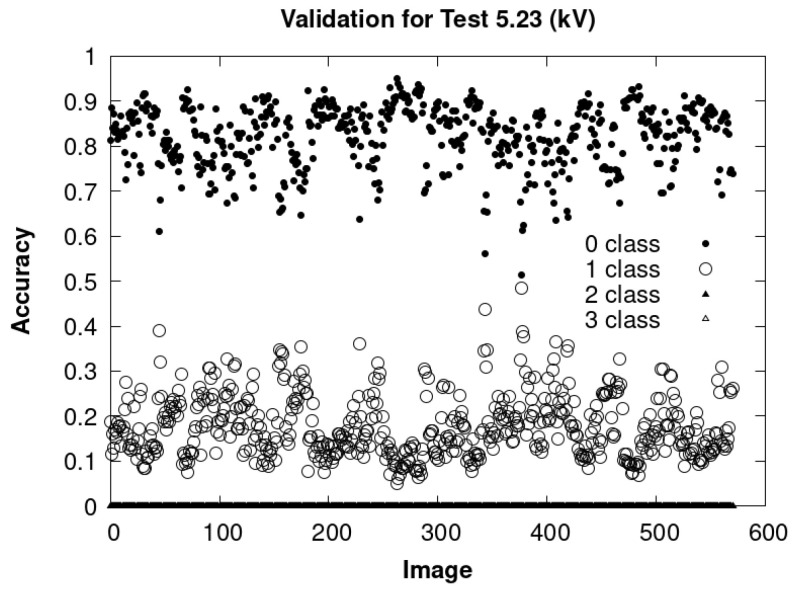
Analysis of 571 consecutive images in a time interval of 19 s. The test was carried out at an effective voltage of 5.23 kV. The neural network classified all images into the class associated with PD with an average accuracy of 0.82.

**Figure 41:**
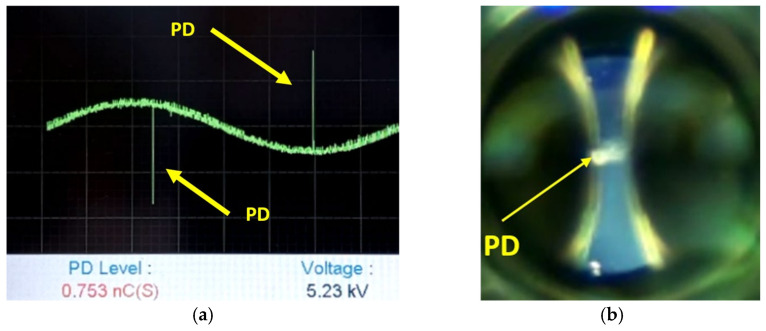
Images corresponding to the initial instant of Figure 40. (**a**) For the DDX-9101 PD detector. (**b**) Taken with the HQ camera.

**Figure 42:**
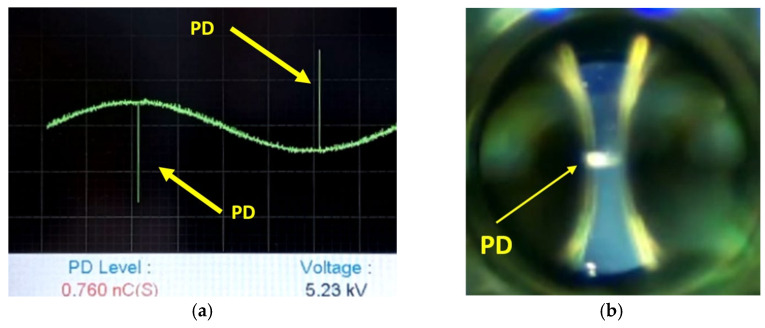
Images of the HQ camera and the PD detector DDX-9101 corresponding to the final instant of the time interval in Figure 41. (**a**) PD measured with the DDX-9101 PD detector at the final instant of the time interval. (**b**) Image taken with the HQ camera at the final instant of the 19 s time interval.

Figure 40 shows the analysis of a video divided into consecutive images, taken during a time interval of 19 s, which corresponds to a total of 571 images. This test was carried out with an effective voltage of 5.23 kV. In this case, the neural network classifies all images as belonging to the class associated with PDs, with an average accuracy of 0.82. Finally, Figure 41 and Figure 42 represent the PD measured with the DDX-9101 PD detector and the image that corresponds to the HQ camera at the initial instant of the time interval and the end, respectively.

These results indicate the ability of the neural network to adequately identify and classify PDs under different conditions and stresses, supporting its effectiveness in detecting this phenomenon.

## 5. Conclusions

In this paper, a non-destructive diagnostic method is developed based on the use of a high-resolution, low-cost CMOS image sensor and the design of a CNN. This method allows information to be obtained about the state of mineral oils before their breakdown occurs, providing a valuable tool for the evaluation and maintenance of these dielectric materials.

An exhaustive experimental analysis of PDs originating from gaseous bubbles present in the dielectric oil was carried out by combining a standard electrical detection system and an optical detection system.

The work presents the numerical results derived from the solution of an equation that describes the behavior of the electric field intensity in a gaseous bubble. These results were compared with those obtained using a known analytical expression, showing that there is an agreement between both, with an error of 2.7%.

A CNN was trained to identify and classify the images captured by the image sensor into four classes: with and without PDs, with arc rupture, and with post-breakdown effects. An image classification model was also developed using deep learning with a CNN based on a TensorFlow and Keras model.

In this work, three different CNN methods based on a TensorFlow were applied: CNN using MobileNetV2, CNN method without dropout, and CNN method with dropout.

The MobileNetV2 method converges the fastest because it is pretrained and there is previous knowledge transfer. Of the other two methods, the one that converges faster is, logically, the one with dropout, since dropout is a regularization strategy widely used in CNN with the purpose of mitigating overfitting.

The performance of the three CNN methods was compared, obtaining an accuracy around 95% in the validation set and 82% in the test set with the best method, which was CNN with dropout.

The reason the accuracy is higher in the validation is that the images used were taken in experiments other than the test ones. The test ones were taken in order to verify the previously trained and validated CNN model.

As already mentioned, the CNN model was trained and validated with a set of 2000 images from the same set of experiments: 80% was used for training and 20% for validation. However, verification of the model was done using 571 images that had not previously been used for training and validation.

## Figures and Tables

**Table 1:** Summary of features of the cameras used.

	V2	HQ
Sensor:	CMOS image sensor Sony IMX219	CMOS image sensor Sony IMX477
Resolution:	3280 × 2464 pixels 8-megapixel	4056 × 3040 pixels 12.3-megapixel
Sensor image area:	3.68 × 2.76 mm 4.6 mm diagonal	6.287 × 4.712 mm 7.9 mm diagonal
Pixel size:	1.12 × 1.12 µm	1.55 × 1.55 µm
Horizontal field of view:	62.2 degrees	Depends on lens
Vertical field of view:	48.8 degrees	Depends on lens
IR cut filter:	Eliminated	Integrated
Back focus length of lens:	3.04 mm	2.6 mm–11.8 mm (M12 Mount) 12.5 mm–22.4 mm (CS Mount)
Substrate material:	Silicon	Silicon

**Table 2:** Experiment ramp of 3 kV/s.

Sample Number		fps		T [°C]	V_rup_ [kV]
	R	C	L		
1	86.0	84.0	83.0	17.2	23.8
2	86.4	85.1	84.0	17.2	21.9
3	87.0	84.6	86.1	16.8	20.5
4	87.0	84.7	86.1	16.8	24.0
5	87.0	84.7	86.3	16.9	24.0
6	87.0	84.7	86.2	16.9	34.3
7	87.0	84.6	86.2	17.1	22.1
8	87.0	84.6	86.0	17.2	24.7

**Table 3:** Experiment ramp of 2 kV/s.

Sample Number		fps		T [°C]	V_rup_ [kV]
	R	C	L		
1	87.1	84.7	86.0	17.3	23.9
2	87.0	84.7	86.2	17.3	22.1
3	87.0	*	86.3	17.5	29.1
4	86.8	84.6	86.3	17.5	35.9
5	87.0	84.7	86.3	17.8	21.4
6	87.0	84.7	86.2	17.7	21.4
7	87.1	84.7	86.2	17.6	31.0
8	87.0	84.6	86.1	17.6	32.8

* Without reading.

**Table 4:** Experiment ramp of 0.5 kV/s.

Sample Number		fps		T [°C]	V_rup_ [kV]
	R	C	L		
1	87.0	84.7	86.2	17.1	24.4
2	87.0	84.7	86.2	17.1	24.4
3	87.0	84.7	86.3	17.1	23.5
4	86.9	84.7	86.2	17.2	23.3
5	87.0	85.0	86.2	17.3	23.1
6	87.0	84.7	86.1	17.3	29.1
7	87.0	84.7	86.1	17.3	30.0
8	87.1	84.7	86.1	17.3	28.0

## Data Availability

Data are contained within the article.
